# A conserved structural framework accompanies evolutionary divergence and expression specialization in the grapevine SKP1 family

**DOI:** 10.3389/fpls.2026.1975054

**Published:** 2026-09-28

**Authors:** Zhen Zeng, Peng Zhao, Xiang-Yun Tian, Lichun Yan, Zhuangwei Wang, Weimin Wu, Wen-Shen Wu, Xicheng Wang

**Affiliations:** 1 Institute of Pomology, Jiangsu Academy of Agricultural Sciences/Jiangsu Key Laboratory for Genetic Improvement and Efficient Utilization of Horticultural Crops, Nanjing, China; 2 State Key Laboratory of Pharmaceutical Biotechnology, School of Life Sciences, Nanjing University, Nanjing, China

**Keywords:** evolutionary diversification, expression specialization, SKP1 family, structural conservation, transcriptional regulation, *Vitis vinifera*

## Abstract

**Introduction:**

S-phase kinase-associated protein 1 (SKP1) proteins are conserved adaptor components of SCF E3 ubiquitin ligase complexes that connect F-box proteins with the CULLIN1 scaffold. Although SKP1-mediated regulation is essential for diverse plant processes, the evolutionary diversification and regulatory specialization of SKP1 family members in grapevine remain poorly understood.

**Methods:**

In this study, genome-wide identification, structural characterization, phylogenetic analysis, promoter analysis, transcriptome profiling, and subcellular localization assays were integrated to investigate the diversification of the grapevine SKP1 family.

**Results:**

Eleven VvSKP1 genes were identified in *Vitis vinifera* and characterized at structural, evolutionary, regulatory, and expression levels. Although individual VvSKP1 members displayed differences in protein architecture and evolutionary relationships, most retained conserved SKP1-like motifs and structural features, indicating preservation of a common molecular framework. Phylogenetic analysis across eight representative angiosperms revealed three major SKP1 lineages and suggested lineage-specific diversification among retained family members. Promoter analyses revealed distinct transcription factor-binding landscapes among VvSKP1 genes, and grapevine TF DAP-seq datasets identified potential MYB-associated regulation of specific members. Transcriptome analyses across diverse grapevine tissues and floral organs revealed expression specialization among VvSKP1 genes, with VvSKP1–9 and VvSKP1–11 showing preferential expression in anther-associated tissues. Despite evolutionary and regulatory divergence, all tested VvSKP1 proteins predominantly localized to the nucleus.

**Discussion:**

These findings reveal a conserved structural framework underlying the grapevine SKP1 family while highlighting diversification in evolutionary relationships, promoter regulatory potential, and tissue-associated expression patterns. This study provides a comprehensive resource for understanding SKP1 family diversification and establishes candidate VvSKP1 members for future functional studies of SCF ubiquitin ligase complexes in grapevine.

## 1 Introduction

S-phase kinase-associated protein 1 (SKP1) is a conserved adaptor protein that links F-box substrate receptors to the CULLIN1 (CUL1) scaffold within SCF (SKP1–CUL1–F-box) E3 ubiquitin ligase complexes. Structural analysis of the CUL1–RBX1–SKP1–SKP2 complex established that SKP1 anchors the F-box substrate-recognition module to the elongated CUL1 scaffold, while more recent studies of assembled SCF complexes have further revealed how this conserved architecture couples substrate recruitment to ubiquitin transfer (Zheng et al., 2002; Horn-Ghetko et al., 2021; Zhu et al., 2024). In plants, structural studies of the Arabidopsis TIR1–ASK1 (Arabidopsis SKP1-like 1) and COI1–ASK1 complexes further demonstrated the conserved role of SKP1-like proteins in assembling hormone-responsive SCF complexes (Tan et al., 2007; Sheard et al., 2010; Tal et al., 2022; Hu et al., 2024). Because individual F-box proteins recruit distinct substrates, the conserved SKP1-centered scaffold supports the assembly of diverse SCF complexes with specialized regulatory functions (Gagne et al., 2002; Kuroda et al., 2012). The plant ubiquitin–26S proteasome system, in which SCF complexes constitute a major class of E3 ubiquitin ligases, regulates the turnover of key proteins involved in hormone signaling, developmental transitions, immune responses, and adaptation to environmental conditions (Vierstra, 2009; Xu and Xue, 2019; Ao et al., 2021; Varshney et al., 2023; Hamada et al., 2026). Given its central role in SCF complex assembly, SKP1 provides an important entry point for understanding the organization and regulatory functions of plant SCF ubiquitin ligase systems.

Across plant lineages, SKP1-like families have expanded through gene duplication and differential retention, generating multiple paralogues with both conserved and lineage-specific features (Kong et al., 2007; Hua and Gao, 2019; Geng et al., 2025). Although many SKP1-like proteins retain core sequence features, individual members have been reported to differ in expression patterns, subcellular localization, or interactions with F-box proteins in a limited number of plant species, pointing to possible member-specific diversification within an otherwise conserved family framework (Kuroda et al., 2012; Shao et al., 2023; Geng et al., 2025). In *Arabidopsis thaliana*, ASK1 and ASK2 exhibit partially redundant functions during embryonic and postembryonic development, whereas ASK1 also has a specialized role in meiotic chromosome organization and male fertility (Liu et al., 2004; Zhao et al., 2006). These observations indicate that SKP1-family evolution cannot be inferred from gene number alone and that protein architecture, regulatory context, and expression patterns should be considered together.

Protein structure provides an additional dimension for examining how conserved molecular functions are maintained during gene-family diversification. Because three-dimensional structure is often more conserved than primary sequence, comparative structural analysis provides a means to test whether a shared family framework coexists with member-specific structural variation (Zhang et al., 2023). Recent advances in AlphaFold-based structure prediction now enable such comparisons across entire gene families, even when experimentally determined structures are unavailable (Abramson et al., 2024). Alongside protein-level diversification, cis-regulatory divergence can contribute to functional specialization among plant gene-family members by reshaping transcription factor-binding potential and generating distinct spatial, developmental, or environmentally responsive expression patterns (Marand et al., 2023). Recent analyses of duplicated genes in soybean further showed that divergence in accessible chromatin regions and cis-regulatory elements is associated with differences in expression level and tissue specificity among retained paralogues (Fang et al., 2023). Integrating promoter regulatory potential with tissue-resolved expression profiles therefore provides a complementary perspective for examining diversification within gene families whose proteins remain structurally conserved.

Grapevine (*Vitis vinifera*) is an economically important perennial fruit crop with increasingly comprehensive genomic resources that facilitate comparative, evolutionary, and functional genomic studies (Shi et al., 2023; Velt et al., 2023; Liu et al., 2024; Wang et al., 2024). As a long-lived woody species with complex tissue organization and developmental programs, grapevine provides an informative system for investigating diversification within conserved regulatory gene families. Although SCF-mediated ubiquitin regulation plays important roles in plant growth, development and environmental responses, the evolutionary relationships, structural conservation, and regulatory specialization of grapevine SKP1 family members remain insufficiently characterized.

Here, we systematically characterized the grapevine SKP1 family by integrating genome-wide identification, gene structure and conserved motif analyses, chromosomal mapping, promoter transcription factor-binding site prediction, phylogenetic reconstruction across eight angiosperm species, transcriptome profiling across grapevine tissues and developmental stages, AlphaFold 3-based structural comparisons, and transient subcellular localization assays. We identified 11 VvSKP1 genes and examined the relationships among their evolutionary grouping, gene organization, promoter regulatory potential, expression patterns, and protein architecture. Despite clear phylogenetic and expression divergence among family members, most VvSKP1 proteins retained a highly conserved structural framework, and all 11 proteins showed nuclear localization in *Nicotiana benthamiana* leaf cells. Together, these results provide an integrated view of structural conservation and regulatory diversification within the grapevine SKP1 family and establish a foundation for functional investigation of individual members.

## 2 Materials and methods

### 2.1 Genome-wide identification and nomenclature of grapevine SKP1 genes

The genome sequences of *Vitis vinifera* (https://phytozome-next.jgi.doe.gov/info/Vvinifera_v2_1, Assembly-version =Genoscope.12X, Annotation-version = v2.1) were searched for SKP1 family members using complementary profile- and similarity-based approaches as described by a previous study (Shao et al., 2014). Briefly, the annotated proteins were searched against the SKP1 domain profile (Pfam accession: PF01466) using hmmsearch implemented in HMMER v3.0 with an E-value cutoff of 1e−4 (Johnson et al., 2010). Candidate sequences were subsequently used as queries for BLASTP searches v 2.16.0+ against the grapevine proteome to identify additional homologous proteins. All candidate sequences were further analyzed using hmmscan against the local Pfam-A database to confirm the presence of the conserved SKP1 domain, with an E-value cutoff of 1e−4. Proteins lacking the conserved SKP1 domain were removed, and the remaining sequences were considered putative grapevine SKP1 proteins. The identified genes were designated VvSKP1–1 to VvSKP1–11 according to their physical positions on grapevine chromosomes.

### 2.2 Gene structure, conserved motifs, and chromosomal distribution

The exon–intron organization of the identified *Vitis vinifera* SKP1 genes was analyzed using the Gene Structure Display Server (Hu et al., 2015). Conserved protein domains and gene structure information were visualized using TBtools (Feng et al., 2026). Conserved motifs among VvSKP1 proteins were identified using the MEME Suite v5.0.5 (Bailey et al., 2015). The chromosomal positions of VvSKP1 genes were obtained from the grapevine genome annotation. Their physical distributions across chromosomes were visualized using the Multiple Genome Viewer (MG2C v2.0; http://mg2c.iask.in/mg2c_v2.0/).

### 2.3 Promoter transcription factor-binding site analysis

The 2,000-bp genomic sequence immediately upstream of each VvSKP1 gene was extracted in a strand-specific manner from the *V. vinifera* genome using the corresponding genome annotation file. Putative transcription factor-binding sites (TFBSs) were predicted using PlantRegMap (https://plantregmap.gao-lab.org/) (Tian et al., 2020). Predicted TFBSs were assigned to their corresponding transcription factor families, mapped along each promoter, and summarized to compare their abundance and distribution among VvSKP1 genes.

To complement the motif-based predictions, published grapevine DNA affinity purification sequencing (DAP-seq) datasets (Orduna et al., 2022) were examined for binding signals associated with the VvSKP1 loci.

### 2.4 Multiple-sequence alignment and phylogenetic reconstruction

All SKP1 family members included in the phylogenetic analysis were independently identified in this study from the proteomes of *V. vinifera*, *Arabidopsis thaliana*, *Oryza sativa*, *Zea mays*, *Glycine max*, *Solanum lycopersicum*, *Populus trichocarpa*, and *Amborella trichopoda* (https://phytozome-next.jgi.doe.gov/) using the genome-wide identification pipeline described above. The full-length amino acid sequences of the identified SKP1 proteins were aligned using ClustalW v2.1 (Thompson et al., 1994). The resulting amino acid alignment was used to reconstruct a maximum-likelihood phylogeny with IQ-TREE 2 v2.4.0 (Minh et al., 2020). The resulting tree topology was visualized and annotated using iTOL v6 (Letunic and Bork, 2024).

### 2.5 Transcriptome data processing and expression profiling

Publicly available normalized expression data representing 54 grapevine tissues and developmental stages were obtained directly from the processed dataset deposited in the Gene Expression Omnibus under accession GSE36128 (Fasoli et al., 2012). Expression values corresponding to the identified VvSKP1 genes were extracted from the normalized expression matrix for subsequent comparison and visualization.

Raw RNA-sequencing reads for the floral-organ dataset were downloaded from NCBI under accession PRJNA736298) (Vannozzi et al., 2021). The dataset included anther, calyptra, calyx, filament, ovary, stigma, and embryo samples collected before anthesis (BA) or after anthesis (AA). The raw reads were processed and quantified using a previously reported transcriptome-analysis workflow (Zeng et al., 2023). Gene expression was normalized as transcripts per million (TPM). The resulting TPM values were used to compare VvSKP1 expression across floral organs and developmental stages.

#### 2.6 Protein structure prediction and structural similarity analysis

Each VvSKP1 amino acid sequence was submitted separately as a single protein chain to the AlphaFold Server, powered by AlphaFold v3 (https://alphafoldserver.com/), using the default settings (Abramson et al., 2024). For each protein, the top-ranked prediction was retained for subsequent analyses. The predicted models were imported into PyMOL v2.5.8, displayed as cartoon representations in a common orientation, and colored according to their predicted local distance difference test (pLDDT) values. Pairwise structural similarity among the 11 grapevine SKP1 proteins was assessed using US-align v20241108 (-mol prot -a T), with TM-scores normalized by the average length of each protein pair.

### 2.7 Subcellular localization assay

To examine the subcellular localization of the VvSKP1 proteins, the coding sequence of each VvSKP1 gene was fused in frame with GFP in the pCHF1 vector, generating C-terminal GFP fusion constructs (primers are listed in **Supplementary Table S1**). Each VvSKP1–GFP construct was co-expressed with the mCherry-tagged Arabidopsis nuclear marker AHL22 (Xiao et al., 2009) in *Nicotiana benthamiana* leaves using *Agrobacterium tumefaciens* strain GV3101-mediated transient expression. At 48 h after infiltration, GFP and mCherry fluorescence signals were observed using an LSM 980 laser-scanning confocal microscope (Carl Zeiss, Germany).

## 3 Results

### 3.1 *Vitis vinifera* genome contains 11 SKP1 genes that are unevenly distributed on the six chromosomes

To identify and characterize the grapevine SKP1 family, HMM-based searches and BLAST analyses were performed against the *Vitis vinifera* reference genome. After removing redundant sequences and confirming the presence of conserved SKP1 domains, 11 non-redundant SKP1-like genes were identified and designated VvSKP1–1 to VvSKP1–11 according to their chromosomal positions (**Figure 1A**; **Supplementary Table S2**). Domain analysis confirmed that all 11 VvSKP1 proteins contained a conserved SKP1 domain, although variations in protein length and terminal regions were observed among family members (**Supplementary Figure S1**).

**Figure 1:**
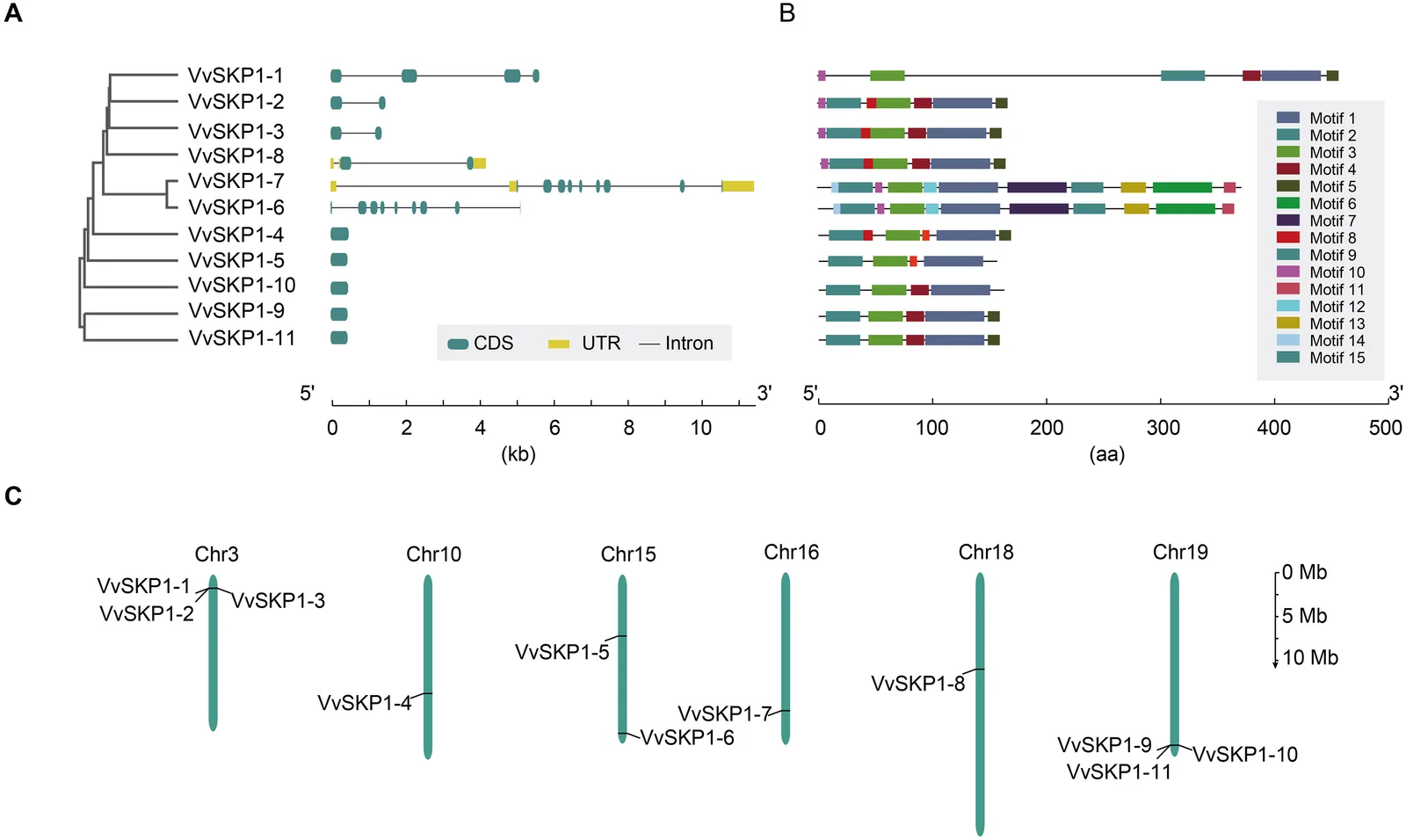
Gene structure, conserved motifs, and chromosomal distribution of the *Vitis vinifera* SKP1 family. **(A)** Family relationships and exon-intron structures of the 11 *VvSKP1* genes. Coding sequences, untranslated regions, and introns are represented by teal boxes, yellow boxes, and black lines, respectively. The phylogenetic tree shown in the left panel was constructed using the maximum-likelihood (ML) method. **(B)** Distribution of 15 conserved motifs in the encoded VvSKP1 proteins. Differently colored boxes represent individual motifs, and the scale indicates protein length in amino acids (aa). **(C)** Physical positions of the *VvSKP1* genes on chromosomes.

To investigate the structural characteristics of VvSKP1 genes, exon–intron organization was analyzed. The gene structures showed considerable conservation among most family members, whereas several genes displayed distinct exon–intron patterns. Specifically, *VvSKP1-4*, *VvSKP1-5*, *VvSKP1-9*, *VvSKP1-10*, and *VvSKP1–11* lacked introns, while the remaining genes contained one or more introns (**Figure 1A**). Among them, *VvSKP1–6* and *VvSKP1–7* exhibited relatively complex gene structures, with *VvSKP1–7* spanning approximately 11 kb genomic region (**Figure 1A**). These variations in exon–intron organization suggest structural diversification among VvSKP1 family members.

The conservation and diversification of VvSKP1 proteins were further examined by analyzing conserved motifs. A total of 15 conserved motifs were identified among the 11 VvSKP1 proteins (**Figure 1B**; **Supplementary Figure S2**). Most VvSKP1 members shared a highly conserved motif arrangement, with motifs distributed in a similar order along the protein sequences, suggesting strong conservation of the SKP1 core region. In contrast, VvSKP1-1, VvSKP1-6, and VvSKP1–7 displayed distinct motif organizations, including altered motif combinations and additional motif regions, which may contribute to the structural diversification of individual family members.

The chromosomal distribution of VvSKP1 genes was examined. The eleven genes were unevenly distributed across six chromosomes, including chromosomes 3, 10, 15, 16, 18, and 19 (**Figure 1C**). Three genes (*VvSKP1-1*, *VvSKP1-2*, and *VvSKP1-3*) were located on chromosome 3, whereas *VvSKP1-9*, *VvSKP1-10*, and *VvSKP1–11* were clustered on chromosome 19. The remaining genes were distributed individually on chromosomes 10, 15, 16, and 18 (**Figure 1C**), revealing an uneven chromosomal distribution pattern of the grapevine SKP1 family.

### 3.2 Predicted VvSKP1 structures reveal a conserved framework with member-specific divergence

To investigate whether the conserved features of VvSKP1 proteins were maintained at the structural level, three-dimensional structures of all 11 VvSKP1 proteins were predicted using AlphaFold3 and evaluated based on pLDDT confidence scores. Most VvSKP1 proteins displayed similar SKP1-like folds, characterized by a conserved structural core despite differences in protein length (**Figure 2A**). The predicted models of VvSKP1-2, VvSKP1-3, VvSKP1-4, VvSKP1-5, VvSKP1-9, VvSKP1-10, and VvSKP1–11 showed relatively high confidence, with average pLDDT values ranging from 81.1 to 89.1 (**Figure 2A**). In contrast, VvSKP1–6 and VvSKP1–7 exhibited lower overall confidence scores (65.1 and 68.8, respectively), mainly due to extended regions with reduced prediction confidence. These results suggest that the conserved SKP1 structural core is maintained among VvSKP1 members, whereas lineage-specific extensions contribute to structural variation in some proteins.

**Figure 2:**
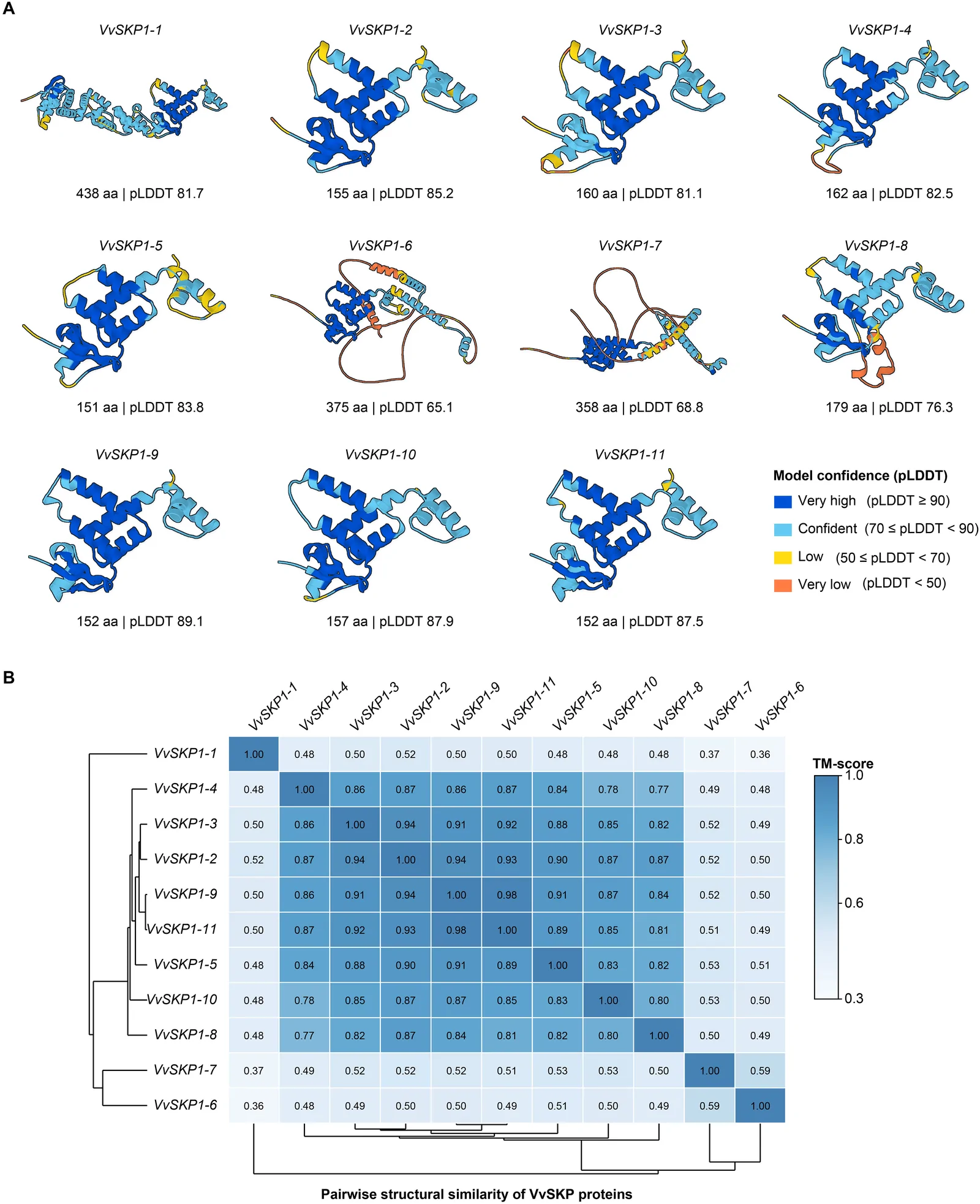
AlphaFold 3-predicted structures and pairwise structural similarity of grapevine SKP1-family proteins. **(A)** Cartoon representations of VvSKP1s in a common orientation after superposition on VvSKP1-1. Structures are colored by pLDDT: dark blue, very high confidence (pLDDT >= 90); cyan, confident (70 <= pLDDT < 90); yellow, low confidence (50 <= pLDDT < 70); and orange, very low confidence (pLDDT < 50). Protein length and mean pLDDT are indicated below each model. **(B)** Clustered heatmap of pairwise structural similarity. TM-scores were calculated using US-align and normalized by the average length of each protein pair. Values in the matrix are pairwise TM-scores; average-linkage clustering used 1 - TM-score as the distance.

To further quantify structural similarity among VvSKP1 proteins, pairwise structural alignments were performed using US-align (**Figure 2B**). Most VvSKP1 proteins exhibited high structural similarity, with TM-scores above 0.8 among the majority of pairwise comparisons, supporting conservation of the overall SKP1 fold. The highest structural similarity was observed between VvSKP1–9 and VvSKP1-11 (TM-score = 0.98), followed by several closely related members including VvSKP1–2 and VvSKP1-3 (TM-score = 0.94). Together, these structural analyses demonstrate that the VvSKP1 family maintains a highly conserved three-dimensional framework while allowing diversification of specific members.

### 3.3 VvSKP1 promoters exhibit distinct cis-regulatory landscapes and candidate MYB regulation of specific members

To investigate the potential transcriptional regulation of VvSKP1 genes, cis-regulatory elements within the 2-kb promoter regions were analyzed using PlantRegMap. The predicted transcription factor binding sites (TFBSs) were distributed throughout the VvSKP1 promoters, with the total number of predicted sites ranging from 31 to 74 among individual members (**Figure 3A**). VvSKP1–7 contained the largest number of predicted TFBSs (74 sites), followed by *VvSKP1-6* (64), *VvSKP1-8* (58), *VvSKP1-5* (57), and *VvSKP1-4* (54), whereas *VvSKP1–9* contained the fewest predicted sites (31) (**Figure 3A**). These differences indicate that individual VvSKP promoters possess distinct cis-regulatory landscapes.

**Figure 3:**
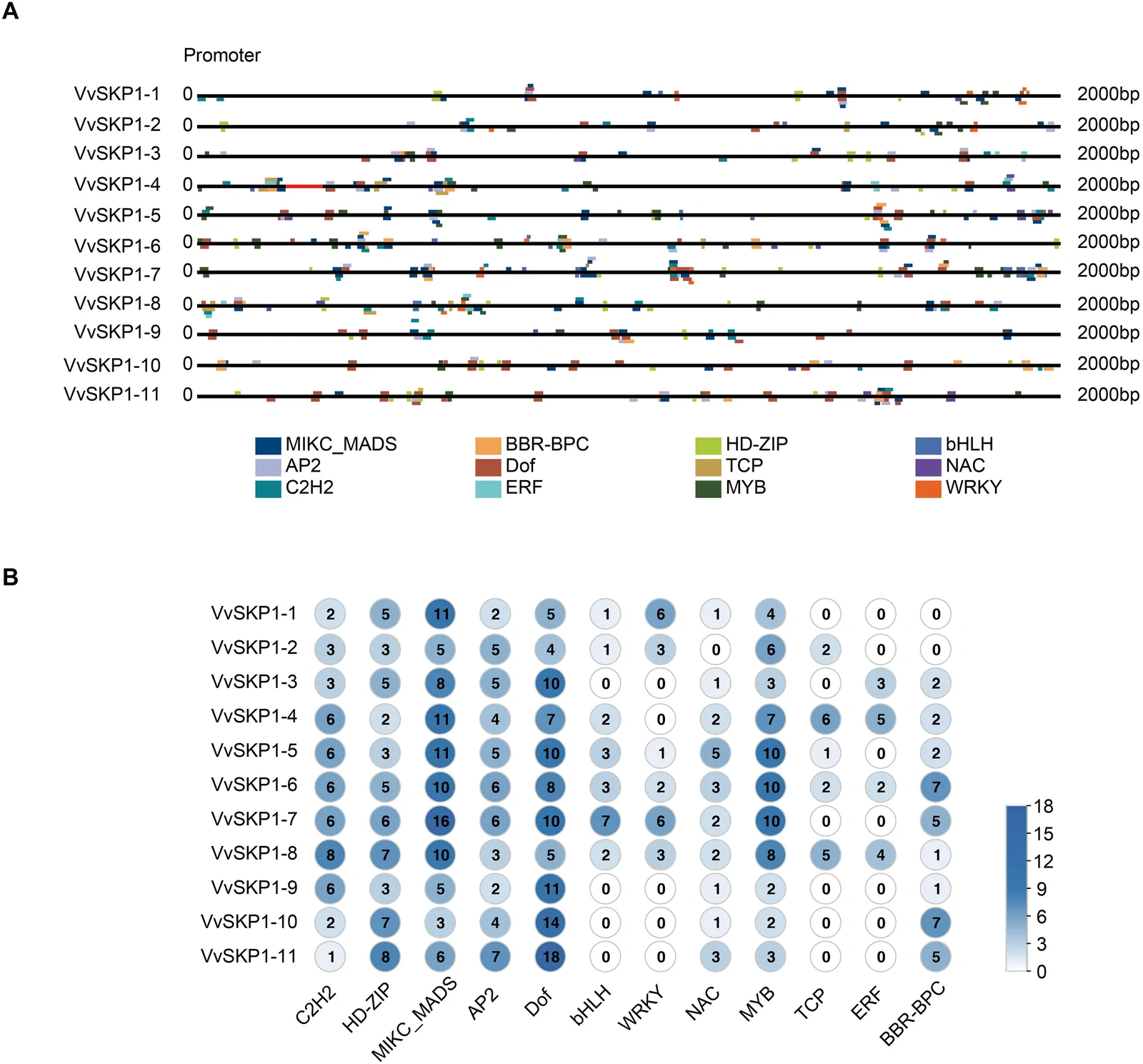
Distribution and abundance of predicted transcription factor-binding sites in *VvSKP1* promoters. **(A)** Locations of 12 TFBS classes within the 2,000-bp upstream region of each gene. Colors denote sites associated with MIKC_MADS, AP2, C2H2, BBR-BPC, Dof, ERF, HD-ZIP, TCP, MYB, bHLH, NAC, and WRKY transcription factors. **(B)** Number of sites in each class. Values within circles give predicted counts, and increasing blue intensity indicates greater abundance.

The composition of predicted TFBSs differed among VvSKP1 promoters. Multiple transcription factor families, including MIKC MADS, Dof, MYB, bHLH, ERF, NAC, and C2H2, were identified within the promoter regions, but their abundance varied substantially among individual genes (**Figure 3B**). For example, *VvSKP1–7* contained the highest number of predicted MIKC_MADS binding sites (16), *VvSKP1–11* contained the highest number of Dof binding sites (18), and *VvSKP1-5*, *VvSKP1-6*, and *VvSKP1–7* each contained 10 predicted MYB binding sites. These differences suggest that individual VvSKP1 genes may be subjected to distinct transcriptional regulatory inputs.

To identify potential transcriptional regulators of VvSKP1 genes, publicly available grapevine transcription factor DAP-seq datasets were systematically screened. This analysis identified three DAP-seq binding events associated with two VvSKP1 loci (Orduna et al., 2022). VvMYB13 showed binding signals at the *VvSKP1–6* and *VvSKP1–7* loci, whereas VvMYB15 showed binding signals at the VvSKP1–7 locus (**Supplementary Table S3**). Notably, *VvSKP1–7* was targeted by both VvMYB13 and VvMYB15, suggesting potential MYB-associated regulatory inputs. Together with the predicted MYB-related cis-elements in VvSKP1 promoters, these results identify MYB transcription factors as candidate regulators of specific VvSKP1 genes.

### 3.4 Angiosperm SKP1 families comprise three major lineages with differential retention and lineage-specific diversification

To investigate the evolutionary history of the VvSKP1 family, a maximum-likelihood phylogenetic tree was constructed using SKP1 homologs from eight representative angiosperm lineages, including the basal angiosperm *Amborella trichopoda*, monocots *Oryza sativa* and *Zea mays*, and diverse dicots *Arabidopsis thaliana*, *Glycine max*, *Populus trichocarpa*, *Solanum lycopersicu*m, and *Vitis vinifera* (**Figure 4A**; **Supplementary Table S4**). The phylogenetic tree resolved SKP1 proteins into three major clades (I–III). The three clades showed distinct taxonomic distributions among the sampled species. Clades I and III included representatives from *A. trichopoda*, monocots, and eudicots, whereas clade II contained monocot and eudicot members but no *A. trichopoda* representative in the present dataset. This pattern may reflect lineage-specific gene loss, sequence divergence, incomplete annotation, or other effects of limited taxon sampling.

**Figure 4:**
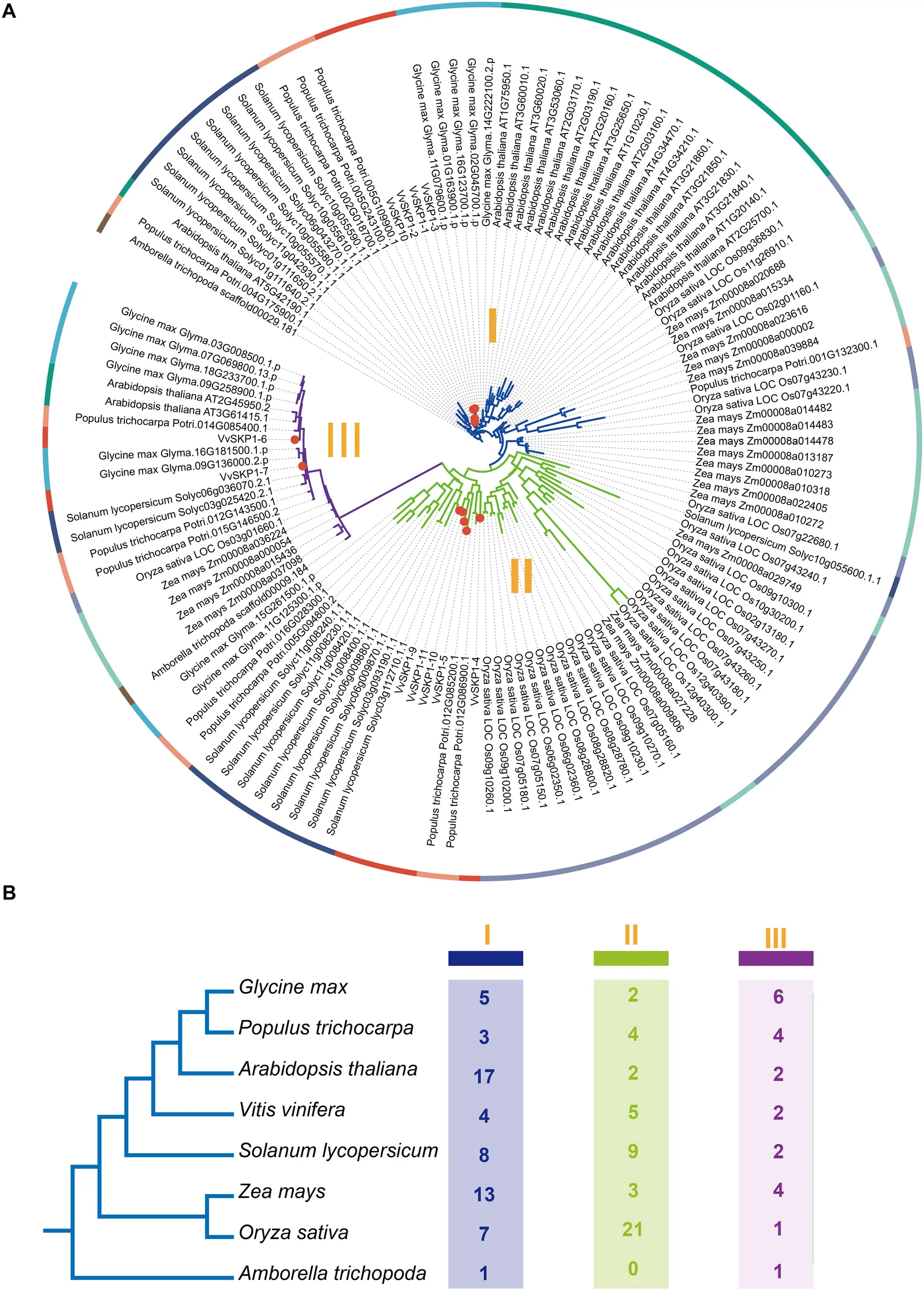
Phylogenetic relationships and group composition of angiosperm SKP1 homologues. **(A)** Maximum-likelihood (ML) tree of SKP1 proteins from *Vitis vinifera*, *Arabidopsis thaliana*, *Oryza sativa*, *Zea mays*, *Glycine max*, *Solanum lycopersicum*, *Populus trichocarpa*, and *Amborella trichopoda*. The colored outer ring indicates species identity, with each color representing a different species. The colored branches distinguish groups I, II, and III, and red circles highlight grapevine proteins. **(B)** Species relationship and distribution of SKP1 homologues among the three groups. Values in the colored columns indicate the number of homologues assigned to each group in each species.

The representation of different plant lineages within each clade was further summarized (**Figure 4B**). Clades I and II were broadly distributed among the sampled species, although the number of retained members varied considerably among lineages. For example, clade I contained more members in *A. thaliana*, *Z. mays*, and *S. lycopersicum*, whereas clade II was particularly expanded in rice and tomato (**Figure 4B**). In contrast, clade III represented a smaller lineage, with fewer members across most species, but was retained in grapevine as VvSKP1–6 and VvSKP1-7 (**Figures 4a, b**). Notably, these two grapevine members showed distinct gene structures and protein features compared with other VvSKP1 proteins (**Figures 1**, **2**), suggesting that they represent a divergent SKP1 lineage within grapevine.

Together, phylogenetic and structural comparisons indicate that the VvSKP1 family consists of conserved evolutionary lineages with lineage-specific diversification patterns across angiosperms.

### 3.5 Expression specialization of VvSKP1 genes across grapevine tissues and floral organs

To investigate expression specialization within the grapevine SKP1 family, transcript accumulation patterns were examined using a comprehensive grapevine tissue expression atlas covering 54 tissues and developmental stages (**Figure 5A**; **Supplementary Table S5**). Ten VvSKP1 genes were represented in the grapevine 54-tissue microarray atlas (Fasoli et al., 2012), whereas *VvSKP1–11* was not included because no corresponding probe was available on the microarray platform. Expression profiles revealed substantial divergence among VvSKP1 members, with individual genes showing distinct tissue-associated expression patterns. For example, *VvSKP1–9* exhibited relatively high expression in stamen in the 54-tissue atlas, whereas *VvSKP1-5*, *VvSKP1-8*, and *VvSKP1–7* displayed relatively higher expression patterns associated with berry-related tissues, vegetative organs, and multiple developmental contexts, respectively (**Figure 5A**). These results indicate that VvSKP1 family members have undergone extensive expression specialization during grapevine development.

**Figure 5:**
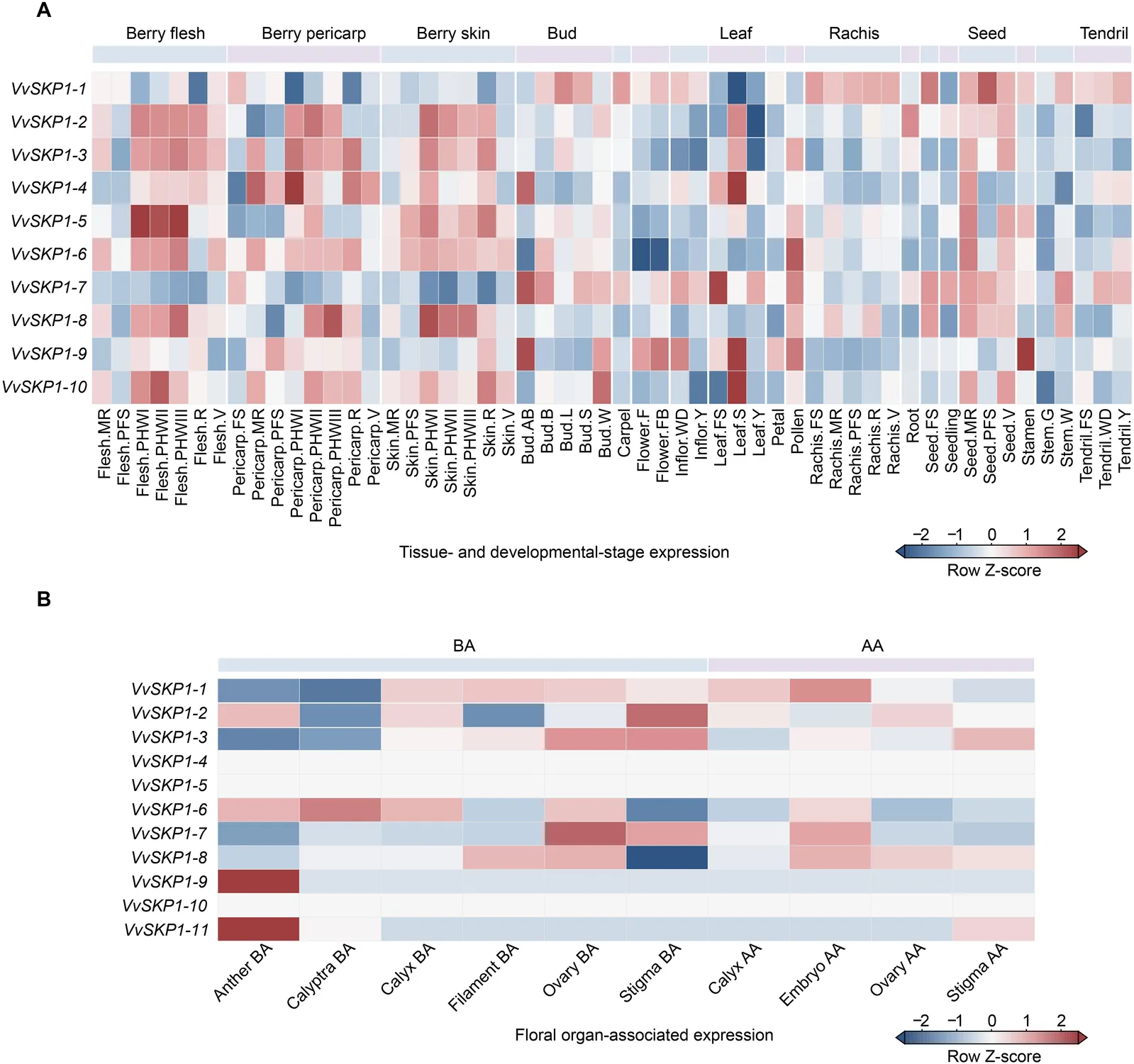
Tissue- and stage-associated relative expression of grapevine SKP1-family genes. **(A)** Row-standardized profiles across 54 tissues and developmental stages from the grapevine expression atlas (GEO GSE36128). *VvSKP1–11* was not included because no corresponding probe was available on the microarray platform. **(B)** Row-standardized profiles across floral organs before anthesis (BA) and after anthesis (AA). Available replicates were averaged, and log2(mean expression + 1) values were converted to row Z-scores. Atlas abbreviations follow Fasoli et al. (2012): AB, after bud burst; B, bud burst; L, latent bud; S, bud swell for buds or senescing for leaves; W, winter for buds or woody for stems; F, flowering; FB, flowering begins; FS, fruit set for berry pericarp, rachis, and seed or mature for leaves and tendrils; G, green; MR, mid-ripening; PFS, post-fruit set; PHWI, PHWII, and PHWIII, first, second, and third months of post-harvest withering, respectively; R, ripening; V, veraison; WD, well developed; Y, young. Stamen and pollen sample names in panel **(A)** follow the original annotations of Fasoli et al. (2012), whereas Anther BA in panel **(B)** denotes anther tissue collected before anthesis. The corresponding unscaled expression values are provided in **Supplementary Table S5**.

To further resolve floral organ-associated expression patterns, an independent floral transcriptome dataset was analyzed (Vannozzi et al., 2021). Consistent with the stamen-associated expression observed in the tissue atlas, *VvSKP1–9* showed preferential expression in anther before anthesis (Anther BA), whereas its expression was relatively low in other floral organs (**Figure 5B**; **Supplementary Table S5**). In addition, VvSKP1-11, which was not represented in the microarray dataset, also showed relatively high expression in Anther BA, suggesting a potential association with male reproductive tissues. Together, these independent transcriptomic datasets highlight *VvSKP1–9* and *VvSKP1–11* as candidate members associated with male reproductive tissues.

### 3.6 VvSKP1 proteins predominantly localize to the nucleus

To determine the subcellular localization of VvSKP1 proteins, the coding sequence of each of the 11 VvSKP1 genes was individually fused with GFP to generate separate VvSKP1–GFP constructs. Each fusion construct was then separately co-expressed with the Arabidopsis nuclear marker AtAHL22–mCherry in *Nicotiana benthamiana* leaves and examined by confocal microscopy (Xiao et al., 2009). The results showed that GFP fluorescence from all 11 VvSKP1–GFP fusion proteins was predominantly concentrated in the nucleus (**Figure 6a**). The nuclear GFP signals closely coincided with the AHL22–mCherry fluorescence, and the overlapping signals were clearly visible in the merged images. This localization pattern was consistently observed for VvSKP1–1 through VvSKP1-11, with no member showing an obviously different subcellular distribution under the experimental conditions used. In contrast, free GFP showed a broad and diffuse intracellular distribution, with fluorescence detectable in both the cytoplasm and nucleus (**Figure 6b**). Thus, despite the sequence and structural variation among individual family members, all 11 VvSKP1 proteins exhibited a broadly similar localization pattern characterized by predominant nuclear accumulation.

**Figure 6:**
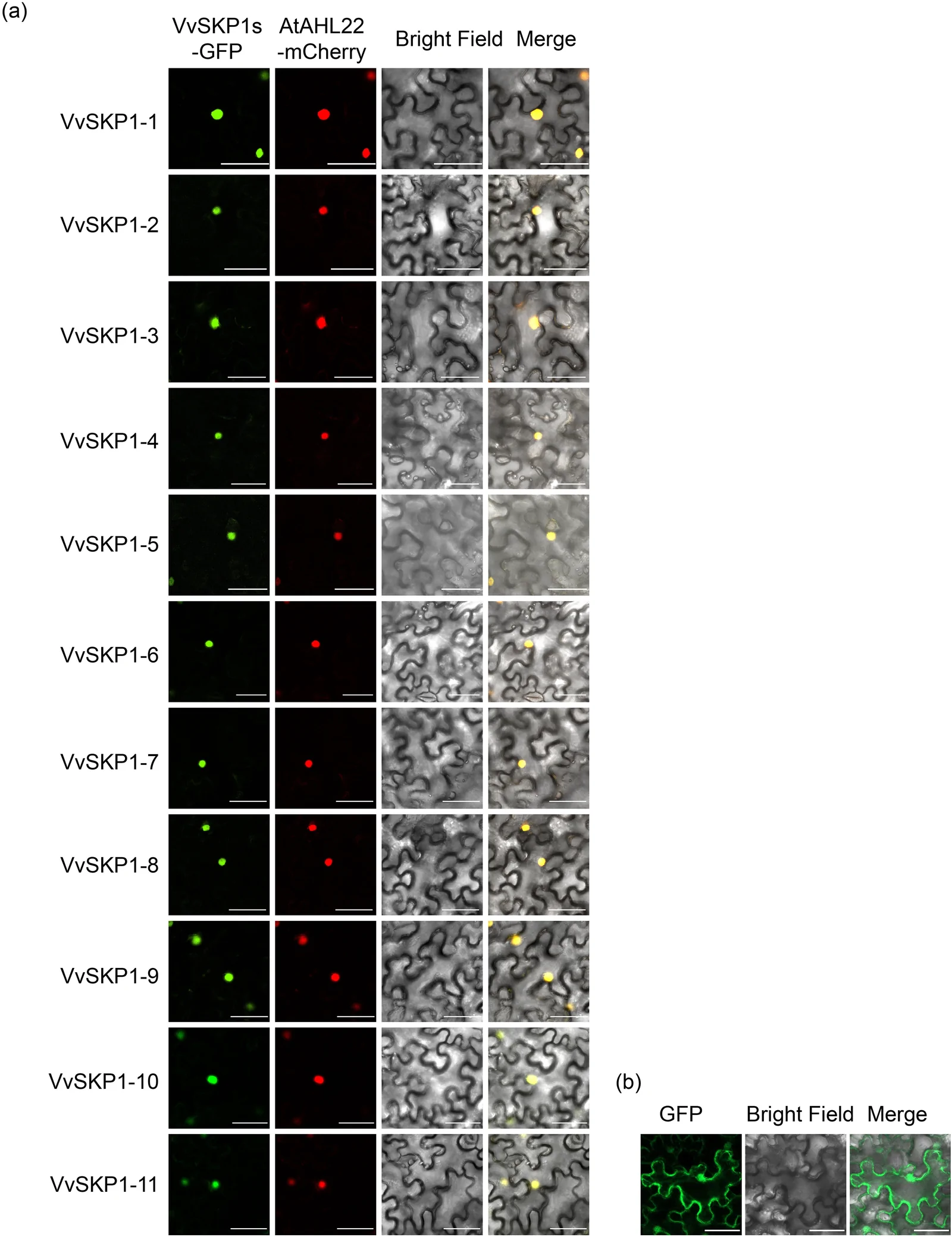
Nuclear localization of the 11 VvSKP1 proteins in *Nicotiana benthamiana*. **(a)** Each VvSKP1 protein was individually fused with GFP and transiently co-expressed with the Arabidopsis nuclear marker AHL22–mCherry in *Nicotiana benthamiana* leaves. Representative fields are shown for the GFP, AHL22–mCherry, bright-field, and merged channels. **(b)** Free GFP was transiently expressed as a control and showed a broad intracellular distribution. Scale bars, 50 μm.

## 4 Discussion

The grapevine SKP1 family represents a conserved adaptor lineage that has maintained a common molecular framework while acquiring member-specific regulatory and expression characteristics. A previous study identified 11 grapevine SKP1 genes and examined their phylogenetic relationships, gene structures, conserved motifs, promoter elements, synteny, and pathogen-responsive expression (Li et al., 2025). Building on this work, we compared SKP1 homologues across eight angiosperms, evaluated the predicted three-dimensional structures of the VvSKP1 proteins, profiled their expression across grapevine tissues and floral organs, and experimentally examined their subcellular localization. Although most VvSKP1 proteins retained a highly conserved SKP1 structural framework and shared nuclear localization, individual members displayed substantial diversification in exon–intron organization, cis-regulatory potential, evolutionary trajectories, and tissue-preferential expression. Notably, integration of phylogenetic, structural, regulatory, and transcriptomic analyses revealed that conservation of the SKP1 core was accompanied by lineage-specific and member-specific specialization. These findings reveal a family-wide pattern in which a conserved structural framework coexists with evolutionary divergence and expression specialization, providing a foundation for functional analysis of individual VvSKP1 members.

The close agreement between motif conservation and predicted three-dimensional structures highlights the strong evolutionary constraint acting on the SKP1 molecular framework. Most VvSKP1 proteins retained highly similar motif organizations (**Figure 1**) and exhibited substantial structural similarity in AlphaFold3 predictions and US-align comparisons (**Figure 2**), indicating that the core SKP1 fold has been maintained during grapevine evolution. In particular, VvSKP1–9 and VvSKP1–11 showed the highest structural similarity among family members, suggesting strong conservation within this lineage. This structural conservation is consistent with the essential role of SKP1 as an adaptor component of SCF ubiquitin ligase complexes, where the conserved scaffold mediates assembly with CUL1 and F-box proteins (Zheng et al., 2002; Horn-Ghetko et al., 2021). Meanwhile, the structural divergence observed in a subset of members, particularly VvSKP1–6 and VvSKP1-7, suggests that evolutionary variation has been accommodated mainly in specific regions outside the conserved SKP1 core.

VvSKP1-1, VvSKP1-6, and VvSKP1–7 represent a structurally divergent subset within the grapevine SKP1 family. Compared with the majority of VvSKP1 members, these proteins contain extended regions and distinct motif organizations, while retaining the conserved SKP1-like framework. Notably, VvSKP1–6 and VvSKP1–7 clustered together in phylogenetic clade III (**Figure 4**), indicating that they constitute a distinct and evolutionarily related lineage within the grapevine SKP1 family. Their lower structural similarity and reduced confidence in peripheral regions indicate that evolutionary variation has preferentially accumulated in non-core regions of the protein scaffold. Such variable regions may provide opportunities for lineage-specific regulation or interaction diversification, but their biological significance will require further experimental validation.

Phylogenetic analysis suggests that diversification of the grapevine SKP1 family is more consistent with the retention of multiple ancestral lineages than with extensive grapevine-specific expansion. Across angiosperms, SKP1-like gene copy number varies substantially as a consequence of lineage-specific duplication, retention, and loss (Kong et al., 2007; Hua and Gao, 2019). Consistent with this broader pattern, grapevine retained a relatively compact repertoire of 11 SKP1 members compared with several species included in our analysis, including *Oryza sativa*, *Zea mays*, and *Arabidopsis thaliana*. The three major clades showed distinct taxonomic distributions across the representative angiosperms (**Figure 4**). The broad representation of clades I and III across *A. trichopoda*, monocots, and eudicots is more consistent with long-term retention of ancient SKP1 lineages than with recent lineage-specific expansion. By contrast, the absence of an *A. trichopoda* representative from clade II suggests a different evolutionary history, potentially involving lineage-specific loss or diversification after the earliest angiosperm splits. Together, these results indicate that grapevine SKP1 evolution has primarily involved maintenance of conserved molecular lineages accompanied by lineage-specific diversification rather than large-scale gene family expansion.

Promoter and expression analyses further suggest that regulatory divergence may contribute to expression specialization among VvSKP1 members. In plant gene families, divergence of cis-regulatory sequences can reshape regulatory landscapes and is frequently associated with differences in expression level and tissue specificity among retained duplicates (Fang et al., 2023). Consistent with this concept, VvSKP1 promoters displayed substantial variation in predicted TFBS composition (**Figure 3**), while transcriptome analyses revealed distinct tissue-associated expression patterns among family members (**Figure 5**). Reanalysis of published grapevine MYB DAP-seq datasets identified VvMYB13 binding signals associated with the *VvSKP1–6* and *VvSKP1–7* loci and VvMYB15 binding signals associated with *VvSKP1-7* (Orduna et al., 2022), suggesting that these genes, particularly *VvSKP1-7*, may receive MYB-associated regulatory inputs.

Among the VvSKP1 members, *VvSKP1–9* provides a prominent example of expression specialization. Its relative expression in the 54-tissue microarray atlas was detected in stamen, and this pattern was independently supported by its relatively high expression in pre-anthesis anthers (Anther BA) in the floral transcriptome dataset. The agreement between two independent datasets supports an association of *VvSKP1–9* with male reproductive tissues. Notably, *VvSKP1–9* belongs to the structurally conserved clade II rather than clade III, which contains the more structurally divergent *VvSKP1–6* and *VvSKP1-7*, suggesting that expression specialization can occur without substantial alteration of the conserved SKP1 protein framework. Studies in *Arabidopsis*, together with more recent family-level analyses in wheat, apple, and cotton, have revealed substantial member-specific variation among SKP1-like paralogues in transcript abundance, developmental expression, subcellular localization, and F-box interaction preferences (Zhao et al., 2003; Dezfulian et al., 2012; Kuroda et al., 2012; Hajsalah El Beji et al., 2019; Geng et al., 2025). *VvSKP1–11* also showed relatively high expression in pre-anthesis anthers in the floral transcriptome dataset, although it could not be evaluated in the microarray atlas because no reliable corresponding probe set was identified on the array platform. These findings identify *VvSKP1–9* and *VvSKP1–11* as candidate SKP1 members associated with male reproductive tissues, although their biological roles remain to be experimentally determined.

At the cellular level, all 11 VvSKP1–GFP fusion proteins showed predominant nuclear accumulation, indicating that the grapevine family retains a shared subcellular context despite divergence in phylogenetic position, promoter architecture, and tissue expression patterns. The potential relevance of this localization is supported by a recent study in rice, in which the SKP1-like proteins OSK1/20 associated with the F-box protein SHPR to form an SCF complex, while SHPR interacted with OsSLK in the nucleus and promoted its polyubiquitination and proteasomal degradation (Ma et al., 2023). Nuclear localization has also been experimentally observed for selected SKP1-like proteins in lemon, whereas SKP1-like proteins in wheat and *N. benthamiana* have shown cytoplasmic, plasma membrane-associated, or dual nuclear–cytoplasmic localization, indicating that subcellular distribution is not universally conserved across plant SKP1 families (Hong et al., 2013; Wang et al., 2025).

Taken together, the evolutionary, structural, regulatory, and expression analyses provide a comprehensive view of VvSKP1 family diversification. Although VvSKP1 proteins retain a conserved structural framework and shared nuclear localization, individual members have acquired distinct regulatory features and tissue expression patterns, suggesting that regulatory specialization contributes to functional diversification while preserving the core SKP1 scaffold. Structurally divergent members, including VvSKP1-1, VvSKP1-6, and VvSKP1-7, further highlight alternative routes of diversification through changes outside the conserved protein core. Future functional studies integrating biochemical interaction analyses and genetic approaches will be important to determine how individual VvSKP1 proteins are incorporated into SCF complexes and how their diversification contributes to grapevine biology.

## 5 Conclusion

The grapevine SKP1 family consists of 11 members that retain a highly conserved SKP1 molecular framework while exhibiting diversification in promoter features and expression patterns. Most VvSKP1 proteins share conserved motif organizations, predicted structural features, and nuclear localization, whereas a subset displays divergent protein features associated with distinct evolutionary lineages. Integrated analyses of promoter composition, phylogenetic relationships, structural characteristics, and tissue expression patterns reveal that conservation of the SKP1 scaffold is accompanied by member-specific regulatory potential and expression specialization. These findings provide an integrated view of how a conserved SKP1 protein family exhibits structural, evolutionary, and expression diversification in grapevine and establish a foundation for future studies investigating the specific biological functions and SCF complex associations of individual VvSKP1 members.

## Data Availability

The original contributions presented in the study are included in the article/**Supplementary Material**. Further inquiries can be directed to the corresponding author.

## Supplementary material

Supplementary Figure 1 Conserved SKP1 domain organization of the 11 VvSKP1 proteins.

Supplementary Figure 2 Conserved motif composition and organization of the 11 VvSKP1 proteins.
