# Supplementary material for: A conserved structural framework accompanies evolutionary divergence and expression specialization in the grapevine SKP1 family
Source: Front Plant Sci. 2026 Sep 28;17:1975054. doi: 10.3389/fpls.2026.1975054 (PMC13655136; doi:10.3389/fpls.2026.1975054)
Supplement: Supplementary file 1 [file Image1.pdf]

Figure S1

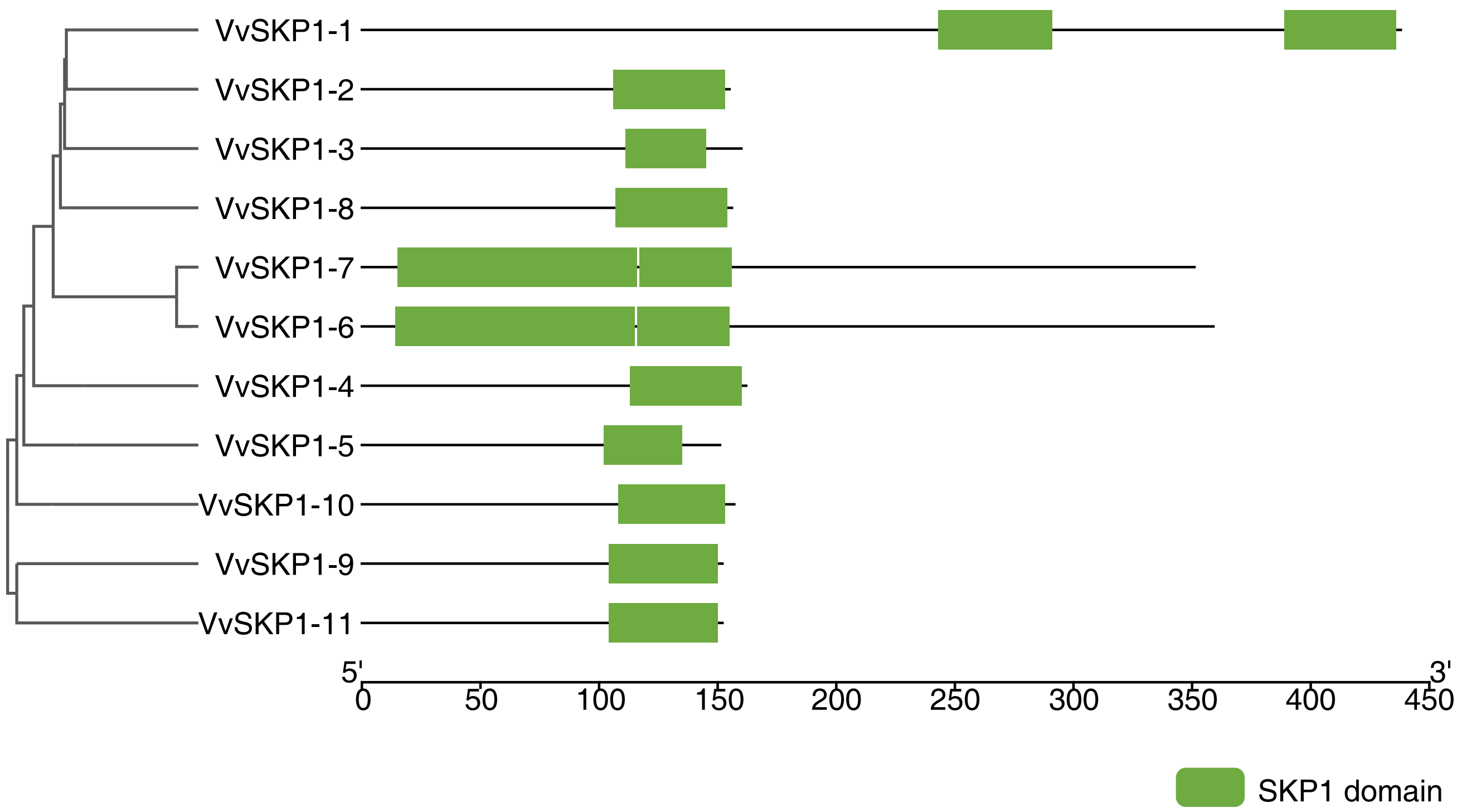
VvSKP1-1
VvSKP1-2
VvSKP1-3
VvSKP1-8
VvSKP1-7
VvSKP1-6
VvSKP1-4
VvSKP1-5
VvSKP1-10
VvSKP1-9
VvSKP1-11
5'
3'
0
50
100
150
200
250
300
350
400
450
SKP1 domain
