# Supplementary material for: A conserved structural framework accompanies evolutionary divergence and expression specialization in the grapevine SKP1 family
Source: Front Plant Sci. 2026 Sep 28;17:1975054. doi: 10.3389/fpls.2026.1975054 (PMC13655136; doi:10.3389/fpls.2026.1975054)
Supplement: Supplementary file 2 [file Image2.pdf]

Figure S2

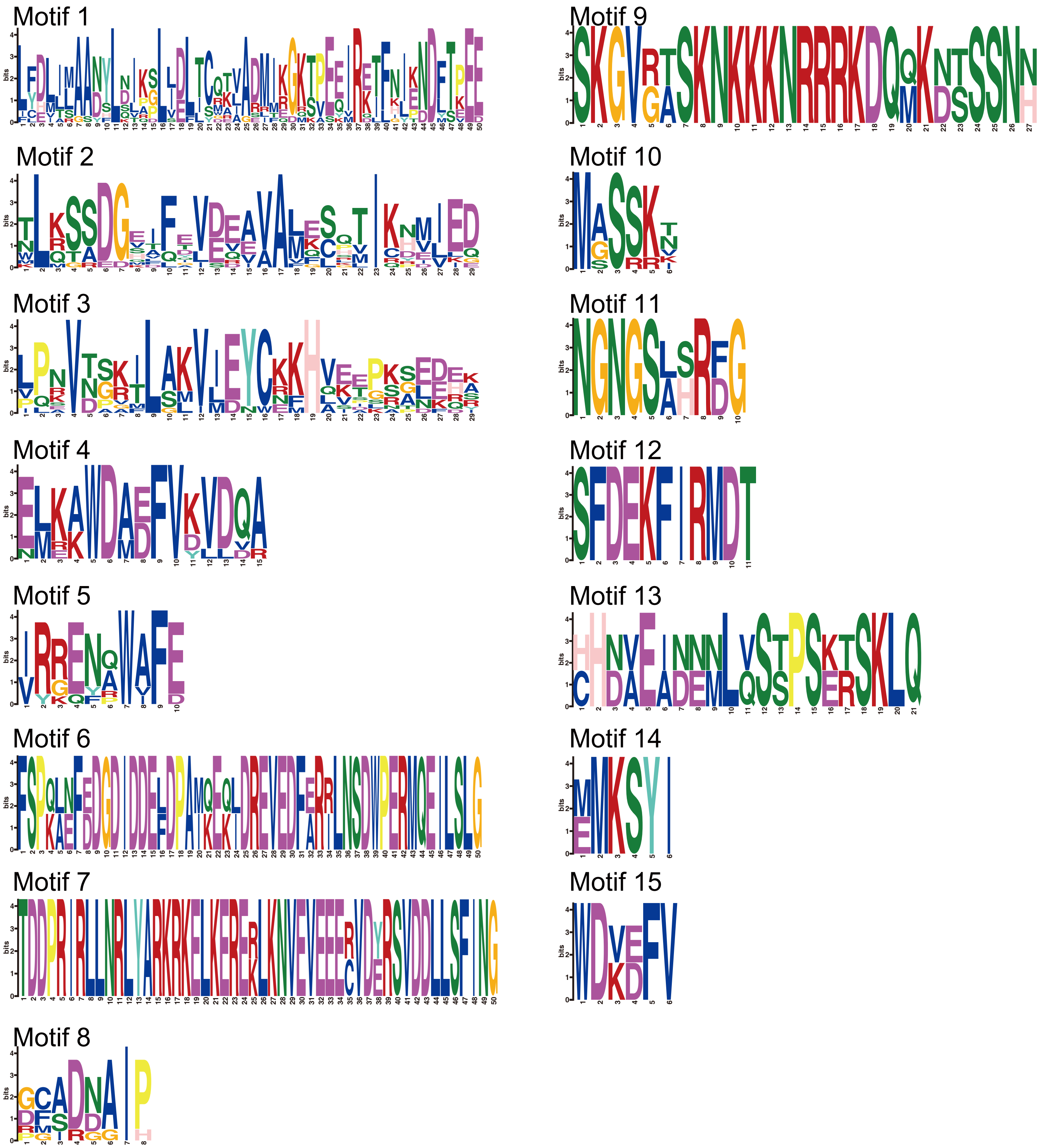
Motif 1
Motif 2
Motif 3
Motif 4
Motif 5
Motif 6
Motif 7
Motif 8
Motif 9
Motif 10
Motif 11
Motif 12
Motif 13
Motif 14
Motif 15
bits
